# Supplementary material for: Molecular Profiling of Inflammatory Mediators in Human Respiratory Syncytial Virus and Human Bocavirus Infection
Source: Genes (Basel). 2023 May 17;14(5):1101. doi: 10.3390/genes14051101 (PMC10218554; doi:10.3390/genes14051101)
Supplement: Supplementary file 1 [file genes-14-01101-s001.zip › genes-2367668-supplementary.pdf]

Table S1. Primers for cytokines and chemokines detection**.**

Primer name	Primer sequence	Reference
IFN-γ -F	5′-TGACCAGAGCATCCAAAAGA -3′	[35]
IFN-γ -R	5′-CTCTTCGACCTCGAAACAGC-3′	
TNF-α-F	5′-CCGAGGCAGTCAGATCATCTT-3′	[36]
TNF-α-R	5′-GCTGCCCCTCAGCTTGA-3′	
IL-1β-F	5′-GGAGCAACAAGTGGTGTTCT-3′	[38]
IL-1β-R	5′-AAAGTCCAGGCTATAGCCGT-3′	
IL 4-F	5′- AACGGCTCGACAGGAACCT-3′	[37]
IL 4-R	5′- ACTCTGGTTGGCTTCCTTCCA-3′	
IL 6-F	5′- ATGTAGCCGCCCCACACAGA-3′	[39]
IL 6-R	5′- CATCCATCTTTTTCAGCCAT-3′	
IL 10-F	5′- TGGGGGAGACCTGAAGAC-3′	[40]
IL 10-R	5′- ACAGGGAAGAAATCGATGACA-3′	
IL 13-F	5′- GGAGCTGGTCAACATCACCC-3′	[41]
IL 13-R	5′- CGTTGATCAGGGATTCCAGG-3′	
IL 8-F	5′- GAGTGATTGAGAGTGGACCACACT-3′	[42]
IL 8-R	5′- GCTCTCTTCCATCAGAAAGCTTTAC-3′	
IL 31-F	5′- ATGATGTACAGAAAATAGTCGAGGAATT-3′	[40]
IL 31-R	5′- CTTCTCTTCCTCCACATCTTTCAAA-3′	
IL 33-F	5′- TGAGACTCCGTTCTGGCCTC-3′	[39]
IL 33-R	5′- CTCTTCATGCTTGGTACCCGAT-3′	
IL 17-F	CATCCATAACCGGAATACCAATA-3′	[43]
IL 17-R	5′- TAGTCCACGTTCCCATCAGC-3′	
IL 18-F	5′- GCTTGAATCTAAATTATCAGTC-3′	[44]
IL 18-R	5′- GAAGATTCAAATTGCATCTTAT-3′	
IL 22-F	5′- GCTTGACAAGTCCAACTTCCA-3′	[45]
IL 22-R	5′- GCTCACTCATACTGACTCCGTG-3′	
IL-1 α-F	5′- ATCAGTACCTCACGGCTGCT-3′	[44]
IL-1 α-R	5′- TGGGTATCTCAGGCATCTCC-3′	
G-CSF-F	5′- AGAGAGTGTCCGAGCAGCAC-3′	[46]
G-CSF-R	5′- CAAGTGAGGAAGATCCAGGG-3′	
CCL2-F	5′- AGGTGACTGGGGCATTGAT-3′	[39]
CCL2-R	5′- GCCTCCAGCATGAAAGTCTC-3′	
CCL3-F	5′- CAT CAC TTG CTG CTG ACA CG-3′	[39]
CCL3-R	5′- TGT GGA ATC TGC CGG GAG-3′	
CCL4-F	5′- CTTCTCTCTCCTCCTGCTTGT-3′	[47]
CCL4-R	5′- GCAAAGGCTGCTGGTCTC-3′	
CCL5-F	5′- CCATGAAGGTCTCCGCGGCAC-3′	[48]
CCL5-R	5′- CCTAGCTCATCTCCAAAGAG-3′	
GM-CSF-F	5′- CACTGCTGCTGAGATGAATGAAA-3′	[49]
GM-CSF-R	5′- GTCTGTAGGCAGGTCGGCTC-3′	
